# *CLPTM1L* Genetic Polymorphisms and Interaction With Smoking and Alcohol Drinking in Lung Cancer Risk: A Case–Control Study in the Han Population From Northwest China

**DOI:** 10.1097/MD.0000000000000289

**Published:** 2014-12-02

**Authors:** Xiaojie Xun, Huijuan Wang, Hua Yang, Hong Wang, Bo Wang, Longli Kang, Tianbo Jin, Chao Chen

**Affiliations:** From the School of Life Sciences, Northwest University, Xi’an, China (XX, HJW, HY, TJ, CC); National Engineering Research Center for Miniaturized Detection Systems, Xi’an, China (HJW, HW, BW, TJ, CC); Key Laboratory of High Altitude Environment and Genes Related to Diseases of Tibet Autonomous Region, School of Medicine, Tibet University for Nationalities, Xianyang, Shaanxi, China (TJ, LK).

## Abstract

Genetic variants of cleft lip and palate trans-membrane 1-like (*CLPTM1L*) genes in the p15.33 region of chromosome 5 were previously identified to influence susceptibility to lung cancer. We examined the association of single nucleotide polymorphisms (SNPs) in *CLPTM1L* genes with lung cancer and explored their potential effects on the relationship between environmental risk factors (smoking, drinking) and lung cancer in a Chinese Han population.

We genotyped 9 single nucleotide polymorphisms (SNPs) of *CLPTM1L* in a case–control study with 228 lung cancer cases and 301 controls from northwest China. Odds ratios (ORs) and 95% confidence intervals (CIs) were calculated by unconditional logistic regression.

We identified that the minor alleles of rs451360, rs402710, and rs31484 in *CLPTM1L* were associated with a 0.52-fold, 0.76-fold, and 0.70-fold decreased risk of lung cancer in allelic model analysis, respectively. In the genetic model analysis, we found rs402710 and rs401681 were associated with decreased lung cancer risk. Further stratification analysis showed that rs380286 displayed a significantly decreased lung cancer risk (OR = 0.65, *P* = 0.041) in the non-drinkers. In addition, Haplotype “GTTATCTGT” was found to be associated with decreased lung cancer risk (OR = 0.50, *P* = 0.033).

Our results verified that genetic variants of *CLPTM1L* contribute to lung cancer susceptibility in the northwest Chinese Han population. Additionally, we found that consumption of alcohol may interact with *CLPTM1L* polymorphisms to contribute to overall lung cancer susceptibility.

## Abstract 2

Supplemental Digital Content is available in the text

## INTRODUCTION

As one of the most common malignant tumors, lung cancer causes the greatest number of cancer-related deaths^1^. The incidence of lung cancer appears to result from a combination of factors, including genetic susceptibility of the individual and risk factors in the environment. Lung cancer has the highest mortality rate, and it is the leading cause of cancer-related deaths in urban China.^2,3^

Several genes at chromosomal locus 5p15.33, including cleft lip and palate trans-membrane 1-like (*CLPTM1L*), were found to be associated with lung cancer risk in recent genome-wide association studies.^4–8^*CLPTM1L*, also known as *CRR9*, encodes an enzyme that may be associated with apoptosis.^9^ This gene is expressed in cells of various tumor types, including lung tumor tissue, and is overexpressed in cisplatin-resistant cell lines.

In modern China, an increasing number of people have smoking and drinking habits. In the northwest China, drinking and smoking is very common. Previous studies have shown that there are interactions between certain genes and environmental factors that increase the risk of lung cancer. But there are few studies reporting an interaction between *CLPTM1L* and environmental factors like drinking and smoking habits. Therefore, in this study, we aimed to determine the relationship between *CLPTM1L* and lung cancer risk, as well as to explore the interaction between *CLPTM1L* and environmental factors. Nine SNPs of *CLPTM1L* were genotyped to perform an association analysis in a case–control study of the northwest Chinese Han population.

## MATERIALS AND METHODS

### Study Participants

All patients and controls were members of the Han population living in Xi’an city and nearby. The patients were recruited between January 2011 and February 2014 at the First Affiliated Hospital of the Medical College of Xi’an Jiaotong University. All patients were newly diagnosed with lung cancer and were characterized histologically. None of the patients had previous history of other cancers, chemotherapy, or radiotherapy. Patients who had comorbidity, such as diabetes mellitus, hypertension, or any endocrine disorders were excluded. The 301 controls were randomly selected from the individuals who received physical examination at First Affiliated Hospital during the same time period as the cases were recruited. The control group comprised unrelated healthy individuals who had no known medical illness or hereditary disorders and who were not taking any medications. Control subjects were excluded if they had <12 months of data before their index date or had miss information. Participants were chosen without restrictions of age, gender, or disease stage. A total of 228 cases and 301 controls were included in the study. The basic characteristics of the participants (eg, gender, age, and pathology) are listed in Table 1.

**TABLE 1:**
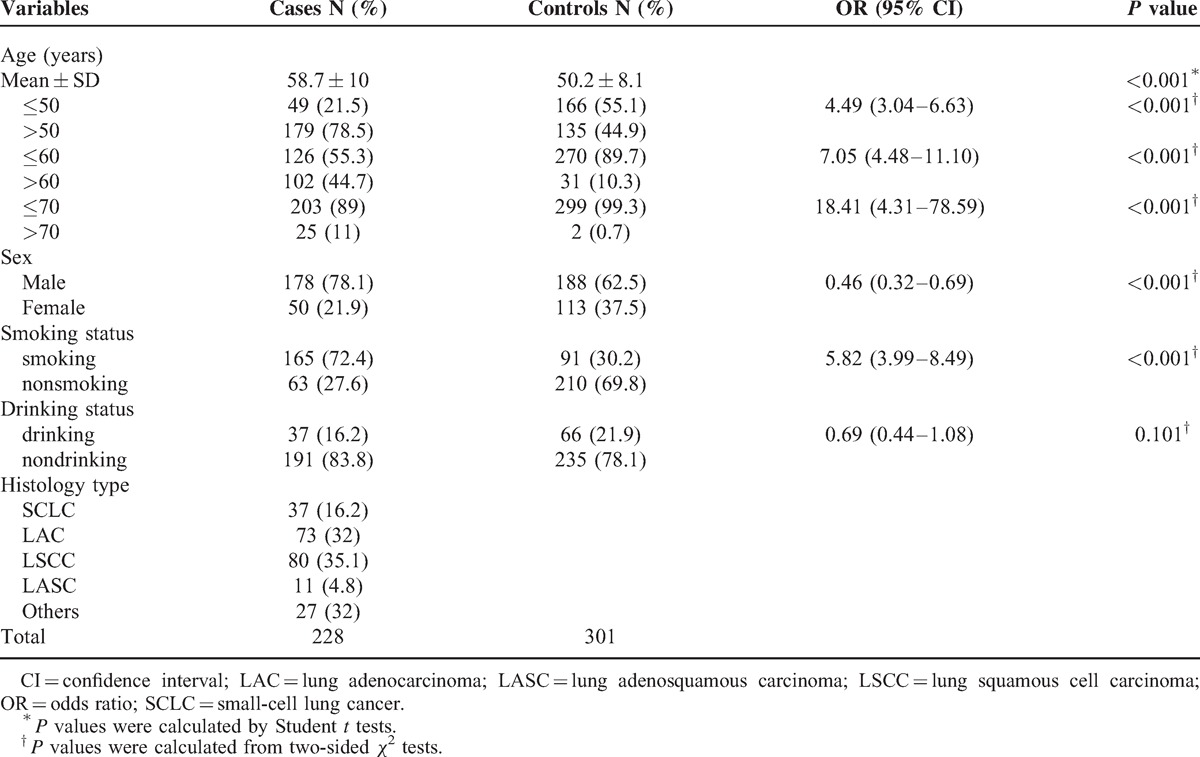
General Characteristics of the Study Population

### Clinical Data and Demographic Information

We used a standard epidemiological questionnaire to collect personal data through an in-person interview, including residential region, age, gender, smoking status, alcohol use, education status, and family history of cancer. Patients were classified as alcohol drinkers if they consumed one or more drinks (Drinking alcohol intake calculated using standard units: one standard drink units = 10 g of pure alcohol = 0.5 two high spirits = 1 two low spirits = 355 mL beer = 1.5 two wine = 3 two wines.) per week, and as nondrinkers if they consumed less than 1 drink per week or abstained from alcohol. All of the smokers had at least 1 or more per day smoking and a period or periods aggregated more than 6 months were defined as ever-smokers; Never-smokers were defined as those who smoked less than 100 cigarettes in their lifetime (or before diagnosis for cases) and former smokers as those who quit smoking at least 1 year before the time of the survey. The case information was collected through consultation with treating physicians or by review of medical charts. All of the participants signed informed consent. The Human Research Committee of the First Affiliated Hospital of Xi’an Jiaotong University and Northwest University for Approval of Research Involving Human Subjects approved the use of human tissue in this study.

### SNP Selection and Genotyping

All 9 SNPs were previously published to be associated with lung cancer, with minor allele frequencies >5% in the HapMap Chinese Han Beijing (CHB) population. The blood samples were collected into tubes containing ethylenediaminetetraacetic acid. After centrifugation, the samples were stored at −80°C until analysis. DNA was extracted from whole blood samples by GoldMag-Mini Whole Blood Genomic DNA Purification Kit (GoldMag Co. Ltd. Xi’an City, China) and DNA concentration was measured by NanoDrop 2000. The Multiplexed SNP MassEXTENDED assay was designed by Sequenom MassARRAY Assay Design 3.0 Software.^10^ SNP genotyping was performed by Sequenom MassARRAY RS1000 using the standard protocol.^10^

### Statistical Analysis

The SPSS17.0 statistical software and Microsoft Excel were used for statistical analysis. All *P* values presented in this study are two-sided, and we used *P* ≤ 0.05 as the cutoff value for statistical significance. We used a Fisher's exact test to assess the departure of each SNP frequency from Hardy–Weinberg equilibrium (HWE) in the control subjects. We tested the difference of SNP genotype distribution between cases and controls by using a χ^2^ test.^11^ We tested odds ratios (ORs) and constructed 95% confidence intervals (CIs) using unconditional logistic regression analysis with adjustments for age, gender, and smoking and drinking status.^12^

The associations between the *CLPTM1L* gene and the risk of lung cancer were tested using genetic models (co-dominant, dominant, recessive, over-dominant and log-additive) analysis by SNP-tats, website software from http://bioinfo.iconcologia.net. We calculated ORs and 95% CIs by unconditional logistic regression analysis adjusted for age and gender.^12^ Akaike's Information Criterion and Bayesian Information Criterion were applied to estimate the best-fit model for each SNP.

Haploview software version 4.2 was used to analyze the association between haplotypes and the lung cancer. Linkage disequilibrium (LD) analysis was performed using genotype data from all the subjects. The pattern of LD was analyzed using two parameters, *r*^2^ and *D*′. Statistical significance was established when *P* < 0.05.

## RESULTS

A total of 529 participants, including 228 lung cancer cases and 301 controls were successfully genotyped for further analysis (Table 1). Males were 78.1% among cases compared with 62.5% among controls. The mean age was 58.7 (±10) years for cases and 50.2 (±8.1) years for controls. There was significant difference between cases and controls as a function of age (≤50 and >50; ≤60 and >60; ≤70 and >70 years old, *P* < 0.001). Overall, there was an increased risk of lung cancer observed with increasing age. More smokers were observed in cases compared with subjects in the control group (*P* < 0.001). This result was expected because most lung cancers can be attributed to smoking. There was no significant difference between groups based upon drinking status (*P* = 0.101). Of the 228 lung cancer cases, 37 (16.2%) were small-cell lung cancer (SCLC), 73 (32%) lung adenocarcinoma (LAC), 80 (35.1%) lung squamous cell carcinoma (LSCC), 11 (4.8%) lung adenosquamous carcinoma (LASC) and 27 (32%) large cell, mixed cell, or undifferentiated carcinomas.

Nine SNPs in the *CLPTM1L* gene were genotyped in lung cancer patients and the healthy controls. Each SNP/sample call rate was 98.5% in both cases and controls. Table 2 summarizes the basic characteristics of *CLPTM1L* SNPs in the study population. rs421629 and rs467095 were excluded at 5% HWE *P* level. Differences in frequency distributions of alleles between cases and controls we determined using the χ^2^ test or Fisher's exact tests. Three SNPs, rs451360, rs402710, and rs31484 were significantly associated with decreased lung cancer risk in the study population, and they respectively presented a 0.52-fold (95% CI, 0.33–0.81, *P* = 0.004), 0.76-fold (95% CI, 0.58–0.99, *P* = 0.045), and 0.70-fold (95% CI, 0.49–0.99, *P* = 0.045) lung cancer risk. After adjustment for age, gender, and smoking and drinking status, the three SNPs respectively presented a 0.59-fold, 0.75-fold and 0.74-fold lung cancer risk.

**TABLE 2:**
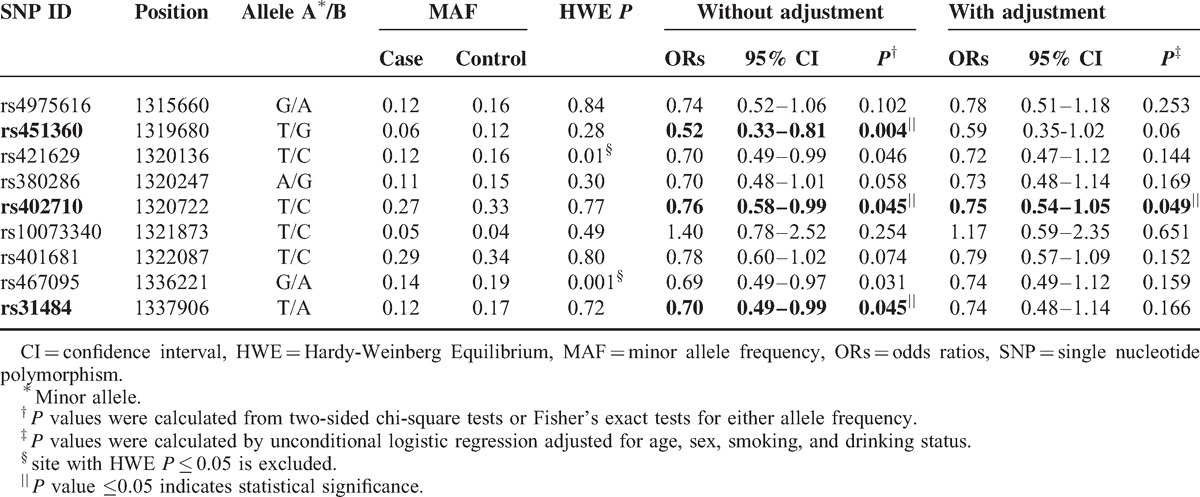
Frequency Distributions of *CLPM1L* Alleles and Their Associations With Lung Cancer Risk

In addition to the allelic model analysis, the association between *CLPTM1L* SNPs and lung cancer risks was assessed using genetic models (Table 3). The results showed that the genotype “T/T” of rs402710 significantly decreased the risk of lung cancer in co-dominant (OR = 0.44; 95% CI, 0.19–0.99, *P* = 0.047) The genotype “T/T” of rs401681 exhibited a decreased risk in the co-dominant model (OR = 0.42; 95% CI, 0.19–0.94, *P* = 0.034) and the recessive model (OR = 0.41; 95% CI, 0.19–0.90, *P* = 0.021).

**TABLE 3:**
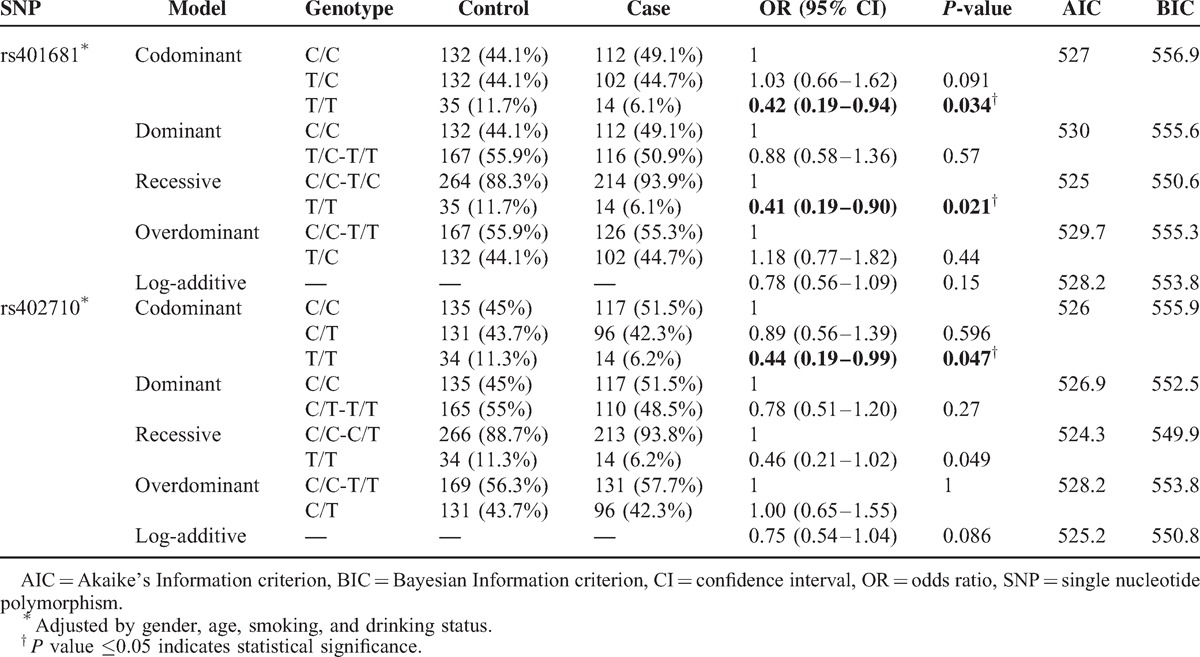
Genotypic Model Analysis of Relationship Between SNPs and Lung Cancer Risk

The association of *CLPTM1L* genetic polymorphisms and lung cancer risk was analyzed among different populations (Supplemental, http://links.lww.com/MD/A103). In the allelic model analysis, rs402710 (OR = 0.55; 95% CI, 0.37–0.81, *P* = 0.002) and rs451360 (OR = 0.50; 95% CI, 0.25–0.969, *P* = 0.04) demonstrated a significantly decreased risk for lung cancer among smokers. After adjustment for age, gender, and drinking status, only rs402710 presented a 0.54-fold decreased risk of lung cancer risk (Table 4). Additionally, rs451360 (OR = 0.43; 95% CI, 0.26–0.72, *P* = 0.001), rs31484 (OR = 0.64; 95% CI, 0.43–0.94, *P* = 0.023), and rs380286 (OR = 0.65; 95% CI, 0.43–0.98, *P* = 0.041) were significantly associated with decreased lung cancer risk in non-drinkers. If consider age, gender and smoking status, rs31484, and rs380286 were not significant anymore (Table 5).

**TABLE 4:**
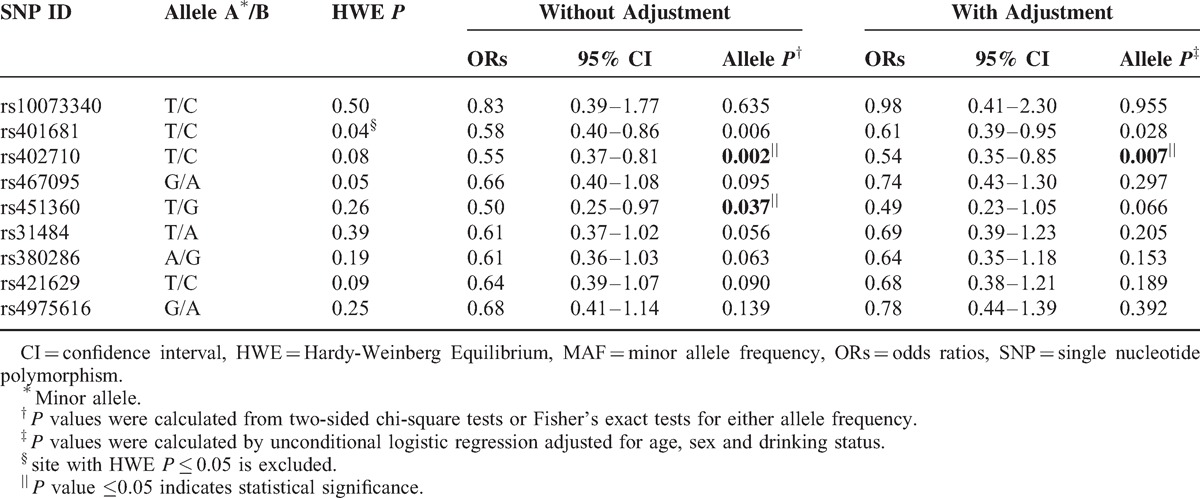
Frequency Distributions of *CLPM1L* Alleles and Their Associations with Lung Cancer Risk in the Smokers

**TABLE 5:**
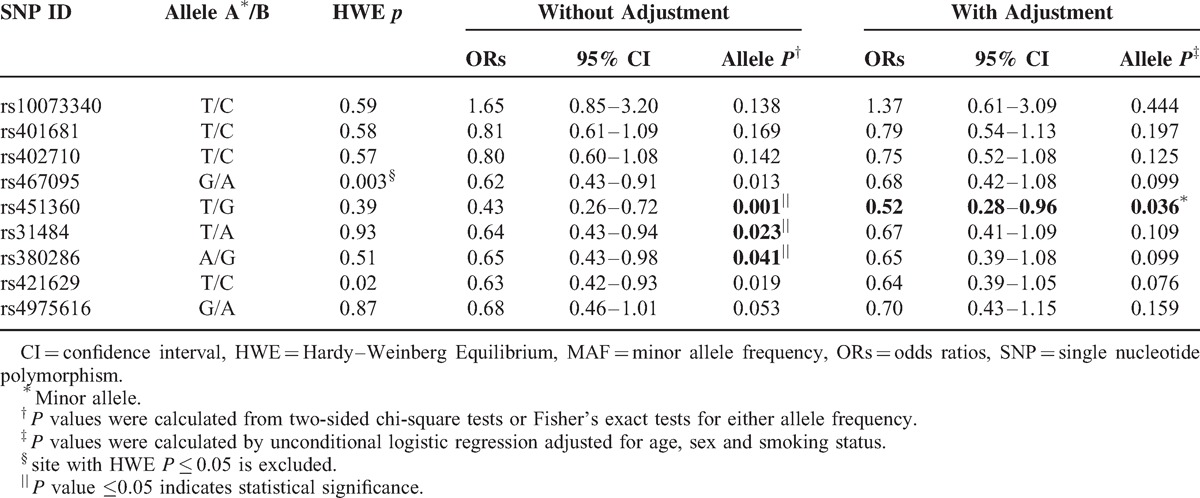
Frequency Distributions of *CLPM1L* Alleles and Their Associations with Lung Cancer Risk in the Nondrinkers

**FIGURE 1:**
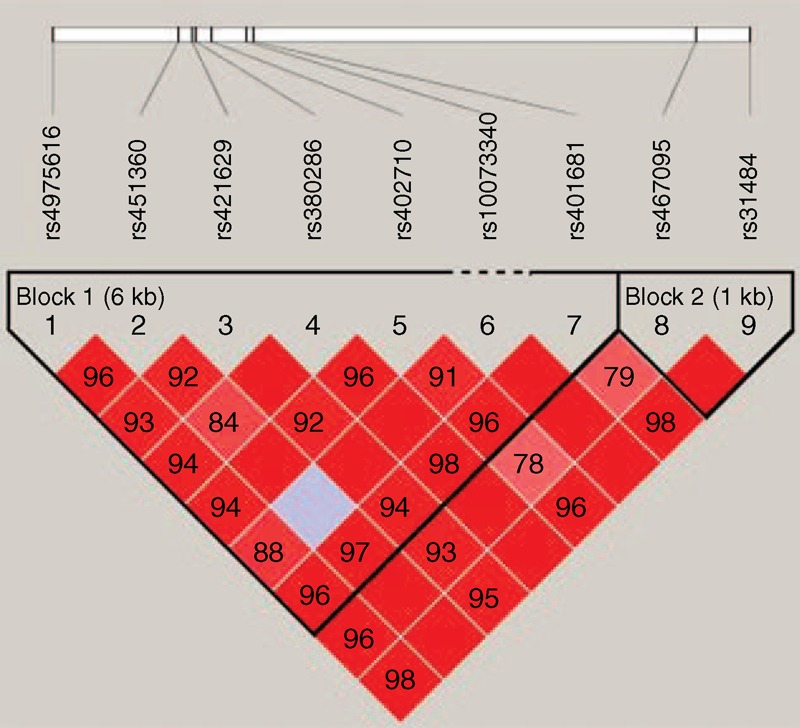
Linkage disequilibrium of polymorphic sites in the *CLPTM1L* gene. A standard color scheme is used to display LD with bright red for very strong LD (LOD = 2, *D*′ = 1), white for no LD (LOD < 2, *D*′ < 1), pink red (LOD = 2, *D*′ < 1), and blue (LOD < 2, *D*′ = 1) for intermediate LD.

*CLPTM1L* polymorphisms were further characterized using linkage disequilibrium (LD) and haplotype analyses. LD was determined pairwise among all 9 SNPs and the haplotype structure of *CLPTM1L* gene was analyzed (*D*′ and *r*^2^). Haplotype block divided by D’ confidence interval method, D’ value of 95% CI 0.70 ∼ 0.98 in adjacent SNPs were classified as the same haplotype block. In the control group, 2 LD blocks were detected. Block 1 consisted of 3 closely linked SNPs, rs421629, rs380286, and rs402710 (Figure 1). The other SNPs in block 1 showed strong linkage. Block 2 included 2 completely linked SNPs, rs467093, and rs31484. Additionally, weak linkage between rs1007334 and rs45136 was observed.

Finally, a haplotype-based association study was performed to show the association between *CLPTM1L* haplotype and risk of lung cancer (Table 6). The haplotype “GTTATCTGT” was associated with decreased lung cancer risk (OR = 0.50; 95% CI, 0.27–0.94, *P* = 0.033).This haplotype was composed by the mutant alleles for rs4975616, rs451360, rs421629, rs380286, rs402710, rs401681, and rs31484, and the reference allele for rs10073340.

**TABLE 6:**
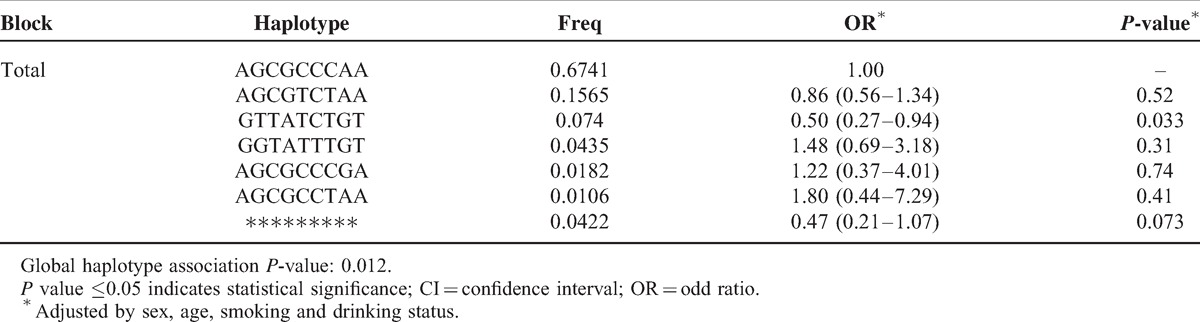
Haplotypes of *CLPTM1L* and Their Association with Lung Cancer Risk

## DISCUSSION

In the present case–control study, we investigated the associations between the 9 SNPs of *CLPTM1L* and risk of lung cancer. We demonstrated that certain *CLPTM1L* genetic polymorphisms, rs402710, rs451360, and rs31484, are associated with the decreased risk of lung cancer in the northwest Chinese Han population. We found that alcohol drinking and tobacco smoking may interact with *CLPTM1L* polymorphisms to affect the development of lung cancer.

*CLPTM1L* is named for its similarity to a gene encoding cleft lip and palate transmembrane protein 1, which was identified as being disrupted in a family with cleft lip and palate.^13^ However, for a long time, the functions of the *CLPTM1L* gene were poorly understood. It was observed as a cisplatin resistance factor in ovarian cancer cell lines, which cannot be interpreted as a function of the *CLPTM1L* gene during oncogenesis.^9,14^ A recent study demonstrated that *CLPTM1L*, as an over expressed protein in lung tumor cells, protected cells from genotoxic stress induced apoptosis through regulation of Bcl-xL, which implicated an anti-apoptotic *CLPTM1L* function as a potential mechanism of susceptibility to lung tumorigenesis.^15^

Of the 3 SNPs associated with decreased lung cancer risk, 2 of these SNPs show high consistency with previous studies in other populations. rs402710, located in intron 4 of the *CLPTM1L* gene, was found to be associated with higher DNA adduct formation in tumor adjacent lung tissue^16^ and may enhance the formation and persistence of DNA adducts. However, the association between this variant and the *CLPTM1L* gene is not clear. The protective effect of rs402710 on lung cancer risk has been identified in epidemiologic studies of the Japanese population,^17^ the Korean population^18,19^ and in a mixed Asian population^20^; however, 1 study of the Chinese population failed to demonstrate a similar finding.^7^ Consistent with most of the previous studies, our study in northwest Chinese Han population confirmed that rs402710 was associated with decreased lung cancer risk in the allelic model. The association between *CLPTM1L* intronic SNP rs451360 and lung cancer has been evaluated in Caucasian^6,21^ and Chinese populations,^4^ which all demonstrated a statistically significant protective effect of rs451360. Consistently, we also found the same effect of rs451360 on lung cancer in the northwest Chinese Han population.

In contrast, rs31484, located in an intron region of *CLPTM1L*, displayed a totally opposite effect on lung cancer risk compared to results of 2 previous studies.^4,21^ One study found rs31484 was associated with an increased lung cancer risk in Caucasians, but the other study showed a decreased risk in Asians. In agreement with the Asian study, we revealed a protective effect of rs31484 in Han Chinese. These conflicting findings might be attributed to the different ethnicity of the subjects enrolled in each study, as the minor allele frequency (MAF) is very different between Caucasians (12%) and Asians (47%).

When subjects were categorized by smoking status, we found that rs402710 and rs451360 were only significant in the smoking group. This result is very interesting because the general belief is that smoking increases the risk of lung cancer. It could be hypothesized that *CLPTM1L* may play a role in apoptotic response of lung cells that are exposed to genotoxic stress caused by tobacco-related carcinogens. However, this hypothesis needs to be investigated in future studies.

It is noteworthy that rs380286 was not associated with lung cancer risk in the general population, but displayed significantly decreased risk for lung cancer among non-drinking population in our study, suggesting it may present a protective effect for non-drinkers to the susceptibility to lung cancer. In one previous study, heavy alcohol consumption was found to be a risk factor for lung cancer.^22–25^ Our data indicated that there may be a potential interaction between *CLPTM1L* polymorphisms and alcohol consumption. However, the role alcohol plays between the *CLPTM1L* polymorphisms and lung cancer risk remains unknown.

Besides the allelic model analysis, we also performed genotypic model analysis to investigate the role of *CLPTM1L* variants on lung cancer risk. We found that in the northwest Chinese Han population, rs401681 and rs402710 had a protective effect on lung cancer in recessive and co-dominant models. Our findings are corroborated by previous studies performed in Asians and Caucasians.^4,18,21,26,27^

Despite the current study possessing enough statistical power, some limitations should be considered. Firstly, the sample size of our study was relatively small. Secondly, the association between *CLPTM1L* polymorphism and clinicopathological type was not evaluated in this study. For example, a meta-analysis showed that rs402710 conferred protective effects in both adenocarcinoma and squamous cell carcinoma.^28^

In conclusion, our study provides new evidence regarding the relationship between *CLPTM1L* and risk of lung cancer in the Han individuals of northwestern China. Our findings suggest that the interaction of genetic variants of *CLPTM1L* and environmental factors, especially tobacco smoking and alcohol consumption, may play an important role in occurrence of lung cancer in this population. The biological functions of the SNPs in *CLPTM1L* are of great interest and warrant further research.

## References

[1] Jemal A Siegel R Ward E Cancer statistics, 2009. *CA Cancer J Clin* 2009; 59:225–249. 19474385 10.3322/caac.20006

[2] Chen W Zheng R Zhang S Lung cancer incidence and mortality in China, 2009. *Thorac Cancer* 2013; 4:102–108. 10.1111/1759-7714.12025 28920188

[3] Chen W Zhang S Zou X Evaluation on the incidence, mortality and tendency of lung cancer in China. *Thorac Cancer* 2010; 1:35–40. 10.1111/j.1759-7714.2010.00011.x 27755783

[4] Hu Z Wu C Shi Y A genome-wide association study identifies two new lung cancer susceptibility loci at 13q12.12 and 22q12.2 in Han Chinese. *Nat Genet* 2011; 43:792–796. 21725308 10.1038/ng.875

[5] Zhong R Liu L Zou L Genetic variations in TERT-CLPTM1L locus are associated with risk of lung cancer in chinese population. *Mol Carcinog* 2013; 52 Suppl 1:118–126. 23908149 10.1002/mc.22043

[6] Pande M Spitz MR Wu X Novel genetic variants in the chromosome 5p15.33 region associate with lung cancer risk. *Carcinogenesis* 2011; 32:1493–1499. 21771723 10.1093/carcin/bgr136 PMC3179422

[7] Jin G Xu L Shu Y Common genetic variants on 5p15.33 contribute to risk of lung adenocarcinoma in a Chinese population. *Carcinogenesis* 2009; 30:987–990. 19369581 10.1093/carcin/bgp090

[8] Wang Y Broderick P Matakidou A Role of 5p15.33 (TERT-CLPTM1L), 6p21.33 and 15q25.1 (CHRNA5-CHRNA3) variation and lung cancer risk in never-smokers. *Carcinogenesis* 2010; 31:234–238. 19955392 10.1093/carcin/bgp287

[9] Yamamoto K Okamoto A Isonishi S A novel gene, CRR9, which was up-regulated in CDDP-resistant ovarian tumor cell line, was associated with apoptosis. *Biochem Biophys Res Commun* 2001; 280:1148–1154. 11162647 10.1006/bbrc.2001.4250

[10] Gabriel S Ziaugra L Tabbaa D SNP genotyping using the Sequenom MassARRAY iPLEX platform. *Curr Protoc Hum Genet* 2009; 2.12.11–12.12.16. 10.1002/0471142905.hg0212s60 19170031

[11] Adamec C Example of the use of the nonparametric test. Test X2 for comparison of 2 independent examples. *Cesk Zdrav* 1964; 12:613. 14246305

[12] Bland JM Altman DG Statistics notes: the odds ratio. *Br Med J* 2000; 320:1468. 10827061 10.1136/bmj.320.7247.1468 PMC1127651

[13] Yoshiura K-i Machida J Daack-Hirsch S Characterization of a novel gene disrupted by a balanced chromosomal translocation t (2; 19) (q11.2; q13.3) in a family with cleft lip and palate. *Genomics* 1998; 54:231–240. 9828125 10.1006/geno.1998.5577

[14] Ni Z Tao K Chen G CLPTM1L is overexpressed in lung cancer and associated with apoptosis. *PloS ONE* 2012; 7:e52598. 23300716 10.1371/journal.pone.0052598 PMC3530437

[15] James MA Wen W Wang Y Functional characterization of CLPTM1L as a lung cancer risk candidate gene in the 5p15.33 locus. *PLoS ONE* 2012; 7:e36116. 22675468 10.1371/journal.pone.0036116 PMC3366984

[16] Zienolddiny S Skaug V Landvik NE The TERT-CLPTM1L lung cancer susceptibility variant associates with higher DNA adduct formation in the lung. *Carcinogenesis* 2009; 30:1368–1371. 19465454 10.1093/carcin/bgp131

[17] Ito H McKay JD Hosono S Association between a genome-wide association study-identified locus and the risk of lung cancer in Japanese population. *J Thorac Oncol* 2012; 7:790–798. 22430809 10.1097/JTO.0b013e3182475028

[18] Yoon K-A Park JH Han J A genome-wide association study reveals susceptibility variants for non-small cell lung cancer in the Korean population. *Hum Mol Genet* 2010; 19:4948–4954. 20876614 10.1093/hmg/ddq421

[19] Bae EY Lee SY Kang BK Replication of results of genome-wide association studies on lung cancer susceptibility loci in a Korean population. *Respirology* 2012; 699–706. 22404340 10.1111/j.1440-1843.2012.02165.x

[20] Hsiung CA Lan Q Hong Y-C The 5p15.33 locus is associated with risk of lung adenocarcinoma in never-smoking females in Asia. *PLoS Genet* 2010; 6:e1001051. 20700438 10.1371/journal.pgen.1001051 PMC2916850

[21] McKay JD Hung RJ Gaborieau V Lung cancer susceptibility locus at 5p15.33. *Nat Genet* 2008; 40:1404–1406. 18978790 10.1038/ng.254 PMC2748187

[22] Li Y Yang H Cao J Association between alcohol consumption and cancers in the Chinese population—a systematic review and meta-analysis. *PLoS ONE* 2011; 6:e18776. 21526212 10.1371/journal.pone.0018776 PMC3078147

[23] Bagnardi V Randi G Lubin J Alcohol consumption and lung cancer risk in the Environment and Genetics in Lung Cancer Etiology (EAGLE) study. *Am J Epidemiol* 2010; 171:36–44. 19933698 10.1093/aje/kwp332 PMC2800301

[24] Bagnardi V Rota M Botteri E Alcohol consumption and lung cancer risk in never smokers: a meta-analysis. *Ann Oncol* 2011; 22:2631–2639. 21427064 10.1093/annonc/mdr027

[25] Genkinger JM Li R Spiegelman D Coffee, tea, and sugar-sweetened carbonated soft drink intake and pancreatic cancer risk: a pooled analysis of 14 cohort studies. *Cancer Epidemiol Biomarkers Prev* 2012; 21:305–318. 22194529 10.1158/1055-9965.EPI-11-0945-T PMC3275675

[26] Rafnar T Sulem P Stacey SN Sequence variants at the TERT-CLPTM1L locus associate with many cancer types. *Nat Genet* 2009; 41:221–227. 19151717 10.1038/ng.296 PMC4525478

[27] Kohno T Kunitoh H Shimada Y Individuals susceptible to lung adenocarcinoma defined by combined HLA-DQA1 and TERT genotypes. *Carcinogenesis* 2010; 31:834–841. 20061363 10.1093/carcin/bgq003

[28] Mocellin S Verdi D Pooley KA Telomerase reverse transcriptase locus polymorphisms and cancer risk: a field synopsis and meta-analysis. *J Natl Cancer Inst* 2012; 104:840–854. 22523397 10.1093/jnci/djs222 PMC3611810

